# Stage 2: The Vaginal Flora in Women Undergoing Fetal Spina Bifida Repair and Its Potential Association with Preterm Rupture of Membranes and Preterm Birth

**DOI:** 10.3390/jcm11237038

**Published:** 2022-11-28

**Authors:** Fanny Tevaearai, Maike Katja Sachs, Samia El-Hadad, Ladina Vonzun, Ueli Moehrlen, Luca Mazzone, Martin Meuli, Franziska Krähenmann, Nicole Ochsenbein-Kölble

**Affiliations:** 1 Department of Obstetrics, University Hospital Zurich, 8091 Zurich, Switzerland; 2 Department of Obstetrics, Faculty of Medicine, University of Zurich, 8006 Zurich, Switzerland; 3 Zurich Center for Fetal Diagnosis and Therapy, 8091 Zurich, Switzerland; 4 Department of Pediatric Surgery, University Children’s Hospital Zurich, 8032 Zurich, Switzerland

**Keywords:** fetal spina bifida (fSB) repair, preterm prelabour rupture of the membranes (PPROM), preterm birth (PTB), vaginal flora

## Abstract

Introduction: Vaginal dysbiosis affects pregnancy outcomes, however, the relevance of abnormal findings on pre/post-surgical vaginal culture in women undergoing fetal spina bifida (fSB) repair is unknown. Objectives: To describe the incidence of normal and abnormal pre- and post-surgical vaginal microorganisms in fSB patients and to investigate potential associations between the type of vaginal flora and the occurrence of preterm prelabour rupture of membranes (PPROM) and preterm birth (PTB). Methods: 99 women undergoing fSB repair were eligible (2010–2019). Pre-surgical vaginal culture was routinely taken before surgery. Post-surgical cultures were taken on indication. Vaginal flora was categorized into four categories: healthy vaginal flora (HVF), bacterial vaginosis (BV), desquamative inflammatory vaginitis (DIV), and yeast infection. Results: The incidence of HVF, BV, DIV, or yeast infections was not statistically different between the pre- and postoperative patients. Furthermore, an abnormal pre/post-surgical vaginal flora was not associated with PPROM (OR 1.57 (0.74–3.32), *p* = 0.213)/OR 1.26 (0.62–2.55), *p* = 0.515), or with PTB (OR 1.19 (0.82–1.73), *p* = 0.315)/(OR 0.86 (0.60–1.24), *p* = 0.425). Conclusions: Abnormal vaginal microbiome was not associated with PPROM and PTB when appropriate treatment was performed.

## 1. Introduction

The vaginal microbiome is a complex combination of multiple bacteria and is known to undergo natural variation during pregnancy [1,2,3,4]. Large studies have shown that vaginal dysbiosis is associated with an increased risk for preterm prelabour rupture of membranes (PPROM) and preterm birth (PTB) [5,6,7]. After open fetal spina bifida (fSB) repair, PPROM and PTB occur in approximately 30–50% and 60–80% [8,9,10], however, no previous study has yet focused on the prevalence of vaginal dysbiosis and its association with PPROM and PTB in the fSB repair population. This study explores the incidence of normal and abnormal pre- and post-surgical vaginal flora and its possible association with PPROM and PTB in fSB patients.

Healthy vaginal flora (HVF) is composed mostly by *Lactobacillus,* which is absent in bacterial vaginosis (BV) and desquamative inflammatory vaginitis (DIV). The flora in BV is dominated by *Gardnerella vaginalis*, whereas in DIV, vaginal inflammation is combined with the presence of *Escherichia coli*, group *B streptococcus*, *Staphylococcus aureus*, or *Enterococcus faecalis* [4].

## 2. Material and Methods

### 2.1. Patient Population and Study Design

One hundred and one pregnant women undergoing open fSB repair from 01/2010-12/2019 at the Zurich Center for Fetal Diagnosis and Therapy were included in this study. Inclusion and exclusion criteria have been adapted from the MOMS (Management of Myelomeningocele Study) criteria and have been described previously [10]. Prenatal closure of the fSB defect was carried out using open fetal surgery. Informed consent was obtained from every patient undergoing fSB repair during pre-operative counseling. Two patients were excluded from the study due to missing informed consent. A total of ninety-nine patients were, therefore, eligible for this study.

Our study was approved by the ethics committee of Canton Zurich (KEK-ZH.Nr. 2015-0172).

The detailed standard admission process on the prenatal ward, as well as intra- and postoperative care at our center, has been described previously [8]. We included the following demographic variables to describe our patient population: maternal age, ethnicity, BMI, smoking, parity, gestational age (GA) at surgery, delivery and birth, cervical length at surgery, fetal gender, and birthweight (Table 1). All patients undergoing open fSB operation received prophylactic antibiotic therapy intraoperative: 1 g of Cefazoline was given iv and 900 mg of Clindamycine was given directly in the amniotic fluid during the operation. In cases of allergy, 500 mg of Vancomycine was given. In the immediate postoperative period, patients were given 1 g of Cefazoline iv 4×/day during 1 day and Clindamycine in cases of allergies.

### 2.2. Vaginal Flora and Treatment

In our cohort, at least three vaginal swabs were taken routinely from all patients before fSB repair at admission to the prenatal ward at 24 + 3/7 GA (20 + 6/7 GA–25 + 5/7 GA).

A wet mount microscopic test was carried out bedside and analyzed by skilled physicians. The vaginal flora was categorized into four adapted categories. Three have recently been established by Paavonen et al. [4]: HVF, BV, and DIV. To these, we added a fourth category, vaginal candidiasis.

A control swab was taken on indication: a control swab in women with a prior abnormal swab after antibiotic therapy, vaginal swabs in case of common clinical symptoms of vaginal infection, and directly after PPROM. These were sent to the laboratory for culture analyses. Whenever the result from the laboratory did not match the diagnosis made by wet mount microscopy, the laboratory results were used.

A third vaginal swab was taken for direct group B streptococcus (GBS)-PCR (GeneXpert, Baden, Switzerland), providing a result of GBS infection status within about 45 min.

Only women with abnormal vaginal bedside swabs were treated promptly with adequate antibiotics before undergoing surgery. If necessary, the antibiotic treatment was adjusted after receipt of the microbiology results.

Postoperatively, vaginal swabs were taken on specific indication: a control swab was repeated in women with a prior abnormal swab after antibiotic therapy to monitor the success of treatment. Vaginal swabs were also performed in case of common clinical symptoms of vaginal infection and directly after PPROM. In the case of PPROM without proof of vaginal dysbiosis, empiric antibiotic treatment was started with erythromycin orally for 7 days and adapted if necessary once culture results were available. After PPROM, a vaginal swab was routinely repeated all 2–3 weeks. In cases with pathological findings, antibiotic treatment was adjusted (or given) according to microbiology results.

BV was treated with dequaliniumchlorid (10 mg vaginally for 6 days), an antimicrobial agent covering Gram-positive and Gram-negative bacteria, anaerobic bacteria, protozoa, and candida. The same treatment was given to women with DIV if no colonization with a resistant germ was found that required other specific treatment. Vaginal candidiasis was treated with fluconazole (150 mg orally, single dose).

## 3. Statistical Analysis

Statistical analysis was performed using the statistical software package SPSS (version 24, IBM, New York, NY, USA). Variables were tested by Kolmogorov–Smirnov test for normal distribution. Quantitative data are presented as mean +/− standard deviation (SD) or medians with IQR. The results of categorical variables are given as percentages.

Odds ratios and 95% confidence intervals were calculated for PPROM and PTB. A *p*-value < 0.05 was considered significant.

## 4. Results

Relevant demographics and clinical variables of the study cohort are shown in Table 1.

**Figure 1:**
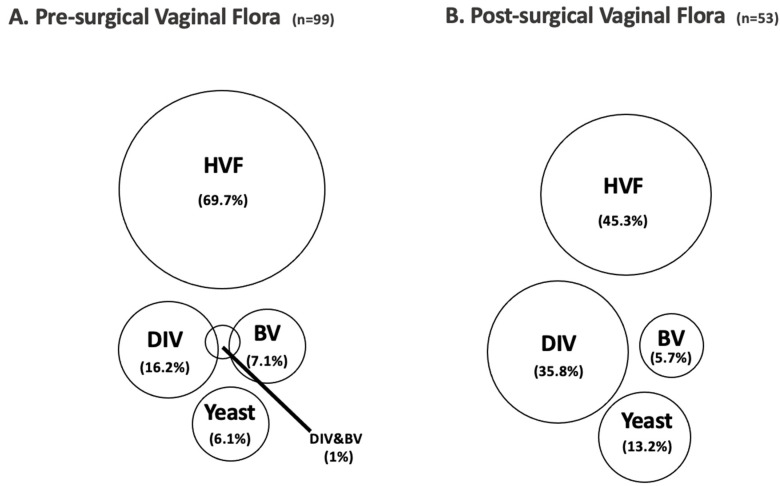
Results of vaginal swabs pre-surgical (**A**) and post-surgical (**B**); HVF—healthy vaginal flora, DIV—desquamative inflammatory vaginitis, BV—bacterial vaginosis.

**Figure 2:**
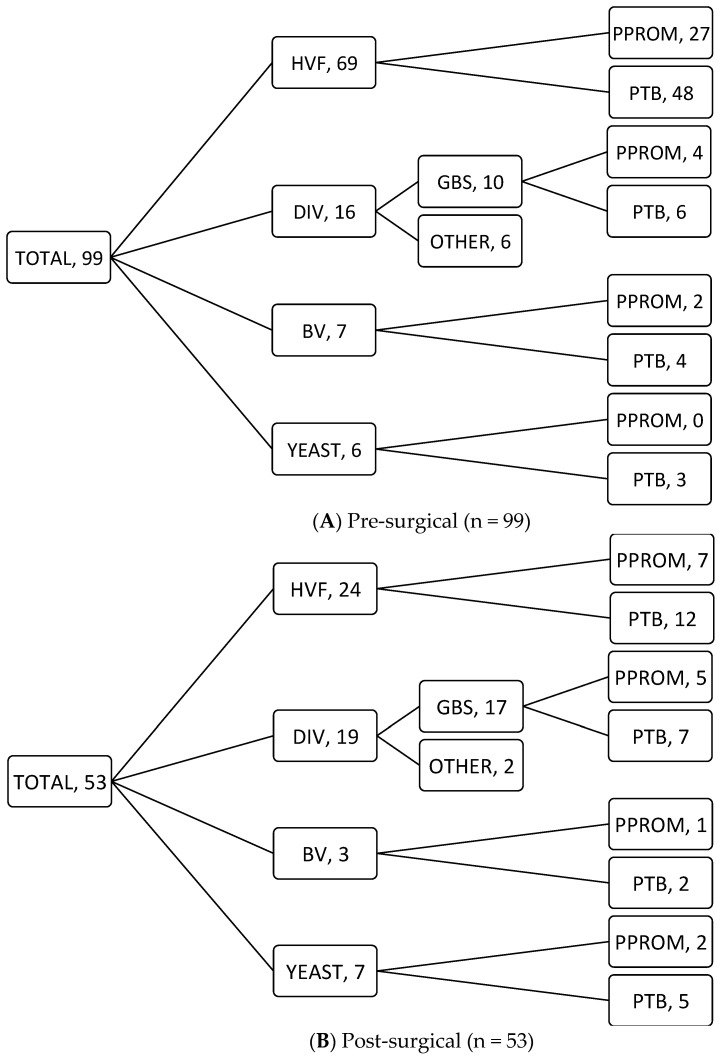
Results of vaginal swabs pre-surgical (**A**) and post-surgical (**B**) and their relation with PPROM and PTB; HVF—healthy vaginal flora, DIV—desquamative inflammatory vaginitis, BV—bacterial vaginosis.

Pre-surgical vaginal swabs were taken routinely from all 99 women: 69 women (69.7%) had a HVF and 30 women revealed an abnormal vaginal flora (30.4%), consisting of 7.1% BV, 16.2% DIV, 1% DIV and BV, and 6.1% yeast infection (see Figure 1A). The relation between these different groups and women ending up having a PPROM vs. PTB is shown in Figure 2A.

Post-surgical cultures were taken in 53 cases on indication: control swab in women with a prior abnormal swab after antibiotic therapy, vaginal swabs in case of common clinical symptoms of vaginal infection and directly after PPROM, while in some women, indications were overlapping. A total of 24 women had a HVF (45.3%), while 29 (54.7%) women showed abnormal vaginal colonization: 3 women with BV (5.7%), 19 women with DIV (35.8%), and 7 women with a yeast infection (13.2%) (see Figure 1B). Once again, the relation between these groups and PPROM vs. PTB is shown in Figure 2B.

An abnormal pre-surgical vaginal flora was not associated with PPROM (OR 1.57 (0.74–3.32), *p* = 0.213) or with PTB (OR 1.19 (0.82–1.73), *p* = 0.315). The same was true for a post-surgical culture where neither PPROM (OR 1.26 (0.62–2.55), *p* = 0.515) nor PTB (OR 0.86 (0.60–1.24), *p* = 0.425) was associated with BV, DIV, or yeast infections.

## 5. Discussion

In our cohort of ninety-nine patients undergoing open fSB repair, there was no association found between abnormal vaginal flora before and after surgery and PPROM or PTB. We found that about two-thirds of women had a HVF before undergoing fSB repair. This is in line with MacIntyre’s study regarding the vaginal microbiome during pregnancy in the general European population, where HVF was found in 66% during pregnancy [3].

As seen in Figure 2, discrepancies appear between the pre- and post-surgical groups. This is probably explained by the fact that post-surgical swabs were only taken on indication. There is, therefore, a selection bias as these indications include women with a prior abnormal swab after antibiotic therapy, women with common clinical symptoms of vaginal infection and directly after PPROM, thus explaining the differences between the two groups. This difference is also explained by the fact that not every infection led to PPROM and/or PTB.

According to previous data [11,12] and in line with this present study, in-utero surgery itself seems to be the major factor causing PTB, most likely as a consequence of direct mechanical membrane damage [13]. Similar to other in utero procedures (i.e., placenta laser [14]), it is presumed that this mechanism is directly linked to PPROM and, therefore, PTB in this population [11,12]. The direct mechanical damage of the membranes puts the woman at a higher risk for chorioamniotic membrane separation and PPROM [15], especially with the earlier timing of surgery [12,16]. Summing up, these studies suggest that PPROM may more likely be due to an iatrogenic effect than it may be induced by an abnormal vaginal flora. However, we cannot exclude that BV, DIV, and yeast infections still represent a risk factor for PPROM and PTB in the fSB repair population as other studies have already shown evidence that vaginal dysbiosis is associated with a higher risk for PPROM and PTB [5,17]. For instance, according to the ACOGs (American College of Obstetricians and Gynecologists) practice bulletin from March 2020, intraamniotic infection was diagnosed in 15–35% of women with PPROM, the incidence being higher in early GA [18]. Although systematic screening and treatment is not recommended in the general low-risk population with asymptomatic BV [19,20,21], studies recommend treating pregnant women with symptomatic BV in order to resolve symptoms [20,22,23]. Regarding pregnant women with BV and a high risk for PTB (i.e., previous PTB or late miscarriage), multiple studies have shown benefits regarding PPROM and PTB when treating BV [24,25]. In Yudin’s study, for instance, treating women with increased risk for PTB was, therefore, recommended [23]. Furthermore, the Oracle I study has shown that an antibiotic treatment in cases of PPROM is evidence-based, hence, decreasing the amount of PTB [26]. In summary, there is evidence that prompt treatment of BV in high-risk situations, such as fSB repair, reduce PTB and PRROM rates.

In cases of vaginal candidiasis, a recent systematic review found no association between asymptomatic vaginal candidiasis and PTB [27]. In other studies, however, treatment of asymptomatic candidiasis is advised in order to prevent PTB [28,29]. As shown in Schusters systematic review, this effect is more likely due to the anti-inflammatory effect of the treatment rather than its anti-fungal treatment [27].

According to the study by Bennett et al., it is assumed that antibiotic treatment destroys the HVF by targeting Lactobacillus and therefore leading to an increased risk for vaginal dysbiosis [5]. This might explain the doubling in yeast infections in our cohort.

As none of these studies focuses on the fSB population, we still think that an appropriate treatment for women with or without symptoms of vaginal dysbiosis or candidiasis may have helped prevent PPROM and PTB in this specific population.

We would like to address a number of limitations and strengths of this study. Our study did not include a control group with patients who were not treated in case of an abnormal vaginal flora. Therefore, we cannot draw any conclusions about whether antibiotic treatment of an abnormal vaginal flora reduced PPROM or PTB.

Another interesting fact to discuss is the antibiotic treatment provided. As described earlier, all women undergoing fSB repair routinely received a prophylactic perioperative antibiotic treatment and no amniotic fluid was collected during the fetal spina bifida repair for bacteriological examination. Recommended treatments for BV include Metronidazole or Clindamycine. Treatments for DIV include Clindamycine or topical glucocorticoid [4]. It is, hence, unknown whether protocol prophylactic antibiotic treatments given to the patients have influenced our results or not. In fact, postoperative vaginal swabs were carried out in the same amount of time, suggesting a possible bias.

A strength of this investigation is the high number of patients of this rather unique study population. In fact, almost all women with PPROM or PTB came back to our center, leading to excellent data quality and completeness. Another interesting aspect of this study is the fact that an evaluation of the vaginal flora was standardized in all patients with an internal control of microscopic diagnosis with the help of an additional swab that was analyzed in the microbiology lab. To our knowledge, other centers do not routinely screen for abnormal vaginal flora and patients are often admitted for delivery to other hospitals than the one that performed the perioperative care.

## 6. Conclusions

In our fSB repair cohort, we found a normal pre-operative distribution of a HVF and there was no association between an abnormal vaginal flora treated antibiotically and PPROM and PTB.

## Figures and Tables

**Table 1:** Demographic and Clinical Variables.

Demographic and Clinical Variables (n = 99)	
Maternal age (years)	32 (26; 35)
Body mass index (kg/m^2^)	25.4 (23; 30)
Smoking	1 (1%)
Nullipara	56 (57%)
Race/Ethnicity (N; %)	
White	93 (94%)
African–American	2 (2%)
Hispanic	2 (2%)
Others	2 (2%)
Cervical length at surgery (mm)	40 (32; 43)
Gestational age at surgery (weeks)	25.0 (24; 26)
Gestational age at delivery (weeks)	36.1 (35; 37)
PPROM	33 (33%)
Preterm birth	65 (66%)
Birthweight (grams)	2650 (2300; 2870)
Female fetal gender	52 (52%)

Data presented as n (%) or median (interquartile range).

## Data Availability

All data generated or analyzed during this study are included in this article. Further enquiries can be directed to the corresponding author.
